# Quantifying the impact of AI recommendations with explanations on prescription decision making

**DOI:** 10.1038/s41746-023-00955-z

**Published:** 2023-11-07

**Authors:** Myura Nagendran, Paul Festor, Matthieu Komorowski, Anthony C. Gordon, Aldo A. Faisal

**Affiliations:** 1 UKRI Centre for Doctoral Training in AI for Healthcare, Imperial College London, London, UK; 2 Division of Anaesthetics, Pain Medicine, and Intensive Care, Imperial College London, London, UK; 3 Brain and Behaviour Lab, Imperial College London, London, UK; 4 Department of Computing, Imperial College London, London, UK; 5 Institute of Artificial & Human Intelligence, University of Bayreuth, Bayreuth, Germany

**Keywords:** Health services, Computer science, Human behaviour, Infectious diseases

## Abstract

The influence of AI recommendations on physician behaviour remains poorly characterised. We assess how clinicians’ decisions may be influenced by additional information more broadly, and how this influence can be modified by either the source of the information (human peers or AI) and the presence or absence of an AI explanation (XAI, here using simple feature importance). We used a modified between-subjects design where intensive care doctors (*N* = 86) were presented on a computer for each of 16 trials with a patient case and prompted to prescribe continuous values for two drugs. We used a multi-factorial experimental design with four arms, where each clinician experienced all four arms on different subsets of our 24 patients. The four arms were (i) baseline (control), (ii) peer human clinician scenario showing what doses had been prescribed by other doctors, (iii) AI suggestion and (iv) XAI suggestion. We found that additional information (peer, AI or XAI) had a strong influence on prescriptions (significantly for AI, not so for peers) but simple XAI did not have higher influence than AI alone. There was no correlation between attitudes to AI or clinical experience on the AI-supported decisions and nor was there correlation between what doctors self-reported about how useful they found the XAI and whether the XAI actually influenced their prescriptions. Our findings suggest that the marginal impact of simple XAI was low in this setting and we also cast doubt on the utility of self-reports as a valid metric for assessing XAI in clinical experts.

## Introduction

AI-driven clinical decision support systems (AI-CDSS) could have a major impact on medical care due to their theoretically super-human performance. In practical settings however, a translation gap remains (especially and counterintuitively within the data-rich environment of critical care medicine) with few systems active in real-world hospital environments^1–3^. This gap implies that the challenge of responsibly guiding clinicians to incorporate AI recommendations into their day-to-day practice might require more than AI suggestions alone. A key demand from clinicians, AI researchers and regulators alike is explainable AI (XAI) which aims to not only provide recommendations but also to justify or motivate the AI reasoning to experts^4,5^. However, most studies that practically evaluate whether and how explanations affect expert decision-making focus on general problems with lessons that do not necessarily translate to high complexity tasks in the clinical sphere^6,7^. In the few cases where medical XAI has been investigated with clinical experts, these have tended to focus on diagnostic scenarios for which a pre-existing gold standard exists with which to calculate accuracy^8–10^. This is not the case for many non-diagnostic medical problems such as the haemodynamic management of sepsis that affects millions of patients worldwide^11^.

Here, we use the flagship example of the AI Clinician system which addresses sepsis resuscitation^12^, a topic fraught with uncertainty, wide variation in clinical practice and no clear optimal solution, at least to the human eye^13,14^. This is despite both decades of research and the provision of international guidelines^15^. The ongoing prospective evaluation of our AI Clinician raises critical questions on how to best render the action recommendations explainable and trustworthy to clinicians who may or may not choose to execute them. This is as much a problem of clinicians’ cognition, influenceability and psychology as one of machine learning^16–18^.

We address these issues in this study by assessing how clinicians’ decisions may be influenced by additional information more broadly, and how this influence can be modified by either the source of the information (human peers or AI) and the presence or absence of an AI explanation (here using simple feature importance). Only by further understanding these critical building blocks for an AI-CDSS can we hope to achieve the end goal of improved outcomes for patients. We find that additional information (peer, AI or XAI) has a strong influence on prescriptions (significantly for AI, not so for peers) but simple XAI does not have higher influence than AI alone. There is no correlation between attitudes to AI or clinical experience on the AI-supported decisions and nor is there correlation between what doctors self-reported about how useful they found the XAI and whether the XAI actually influenced their prescriptions. Our findings suggest that the marginal impact of simple XAI is low in this setting and we also cast doubt on the utility of self-reports as a valid metric for assessing XAI in clinical experts.

## Results

86 ICU doctors were recruited (31 senior [consultant/attending], 42 intermediate [registrar/fellow], 13 junior [senior house office/resident]). Median subject age was 37 years (interquartile range (IQR) 34–43). Median years of clinical experience was 11 years (IQR 9–19). All subjects completed the task successfully and there was no significant difference in per trial completion time between arms (baseline 83 seconds (s), peers 83s, AI 79s, XAI 83s, *p* = 0.574 by Kruskal–Wallis test).

### Impact of arms on dosing shift

**Fig. 1:**
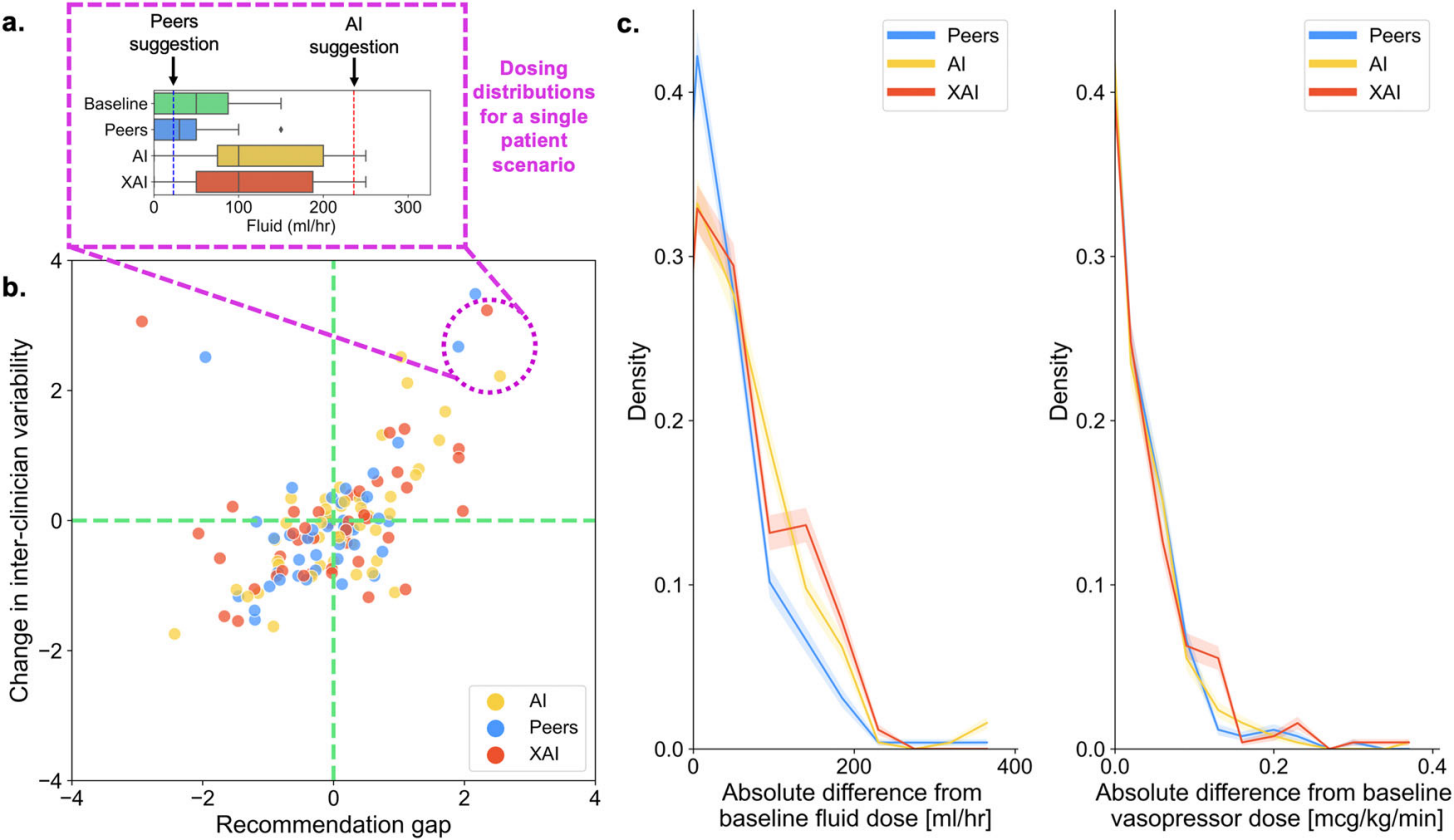
Dose shift and variability by intervention arm. **a** The prescription distributions for a single patient scenario (and, for illustrative purposes only, mapped onto (**b**)). For each boxplot, the centre line represents the median, box edges represent upper and lower quartiles, whiskers represent 1.5× inter-quartile range and diamonds are outliers. Blue dashed line represents the median of the peer distribution data (only available to those in the ‘Peers’ arm). Red dashed line represents the AI suggested dose (only available to those in the ‘AI’ or ‘XAI’ arms). 2b, change in inter-clinician variability by size of recommendation difference for peers/AI/XAI (i.e. was the recommended dose higher (positive recommendation gap) or lower (negative recommendation gap) than the baseline average (dashed green line) and how does this affect variability of clinicians (*x*- and *y*-axes scales are arbitrary units, normalised to allow fluid and vasopressor to be plotted together. **c** Absolute difference (i.e. 50 ml in either direction treated as +50 ml discordance) from dose in the baseline group, aggregated for all 24 patient scenarios. The error bars are formed by randomly taking 1000 bootstraps of the data (80% subset with replacement) and estimating a distribution for prescription doses (error bar is distribution mean +/− standard deviation).

An example of prescription shift for an individual patient scenario is shown in Fig. 1a. For the same patients in different arms, providing subjects with additional information from their respective arm led to an absolute prescription shift for fluid of 70 mls/hr (peers, standard deviation (SD) 86 mls/hr), 90 mls/hr (AI, SD 83 mls/hr) and 85 mls/hr (XAI, SD 60 mls/hr) relative to the baseline arm (*p* = 0.872 for peers, *p* = 0.002 for AI, *p* = 0.007 for XAI, all by independent *T*-test). For vasopressor, the prescription shift was 0.04 mcg/kg/min (peers, SD 0.06 mcg/kg/min), 0.05 mcg/kg/min (AI, SD 0.09 mcg/kg/min) and 0.05 mcg/kg/min (XAI, SD 0.09 mcg/kg/min) relative to the baseline arm (*p* = 0.201 for peers, *p* = 0.010 for AI, *p* = 0.002 for XAI, all by independent *T*-test). The aggregate prescription shifts are displayed in Fig. 1c. The individual patient scenario dosing shift figures for all 24 patients are shown in Supplementary Fig. 1.

### Impact of arms on practice variation

Providing doctors with a recommendation (be it peer, AI or XAI) had a common effect: inter-clinician dose variability was differentially affected according to whether the recommendation was higher or lower than what subjects in the baseline arm did, i.e., when the recommendation was higher than baseline, the prescriptions of doctors in the peer/AI/XAI arms would be more variable across doctors; when it was lower than baseline, prescriptions were less variable across doctors. This can be seen in Fig. 1b.

### Association of clinician factors with adherence to AI suggestions

**Fig. 2:**
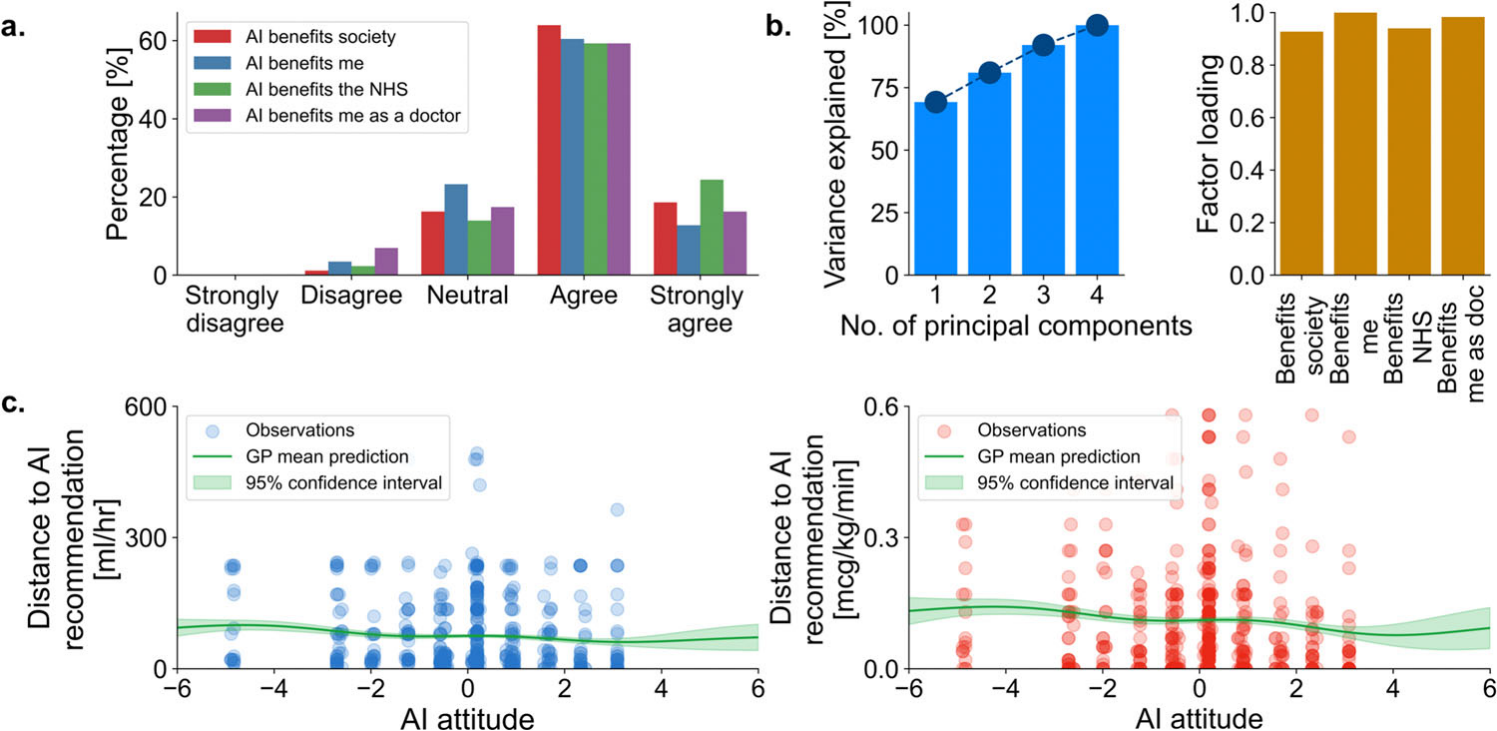
Impact of AI attitude on adherence to AI suggestions. Four AI statements were presented to subjects pre-experiment who were asked for their agreement (**a**). Principal component analysis was applied to the results of these four questions with 69% of variance explained by a single component (**b**). This single component formed our composite for AI attitude (higher value, more positive AI attitude), which was then compared to absolute difference from the AI suggested dose, i.e., a proxy for AI adherence with lower value indicating greater adherence and vice versa in (**c**) for both fluid (blue) and vasopressor (red). Dot transparency in 3c represents density of points at any given location. ‘GP mean’ refers to a predicted Gaussian Process regression fit of the data with accompanying 95% confidence interval.

**Fig. 3:**
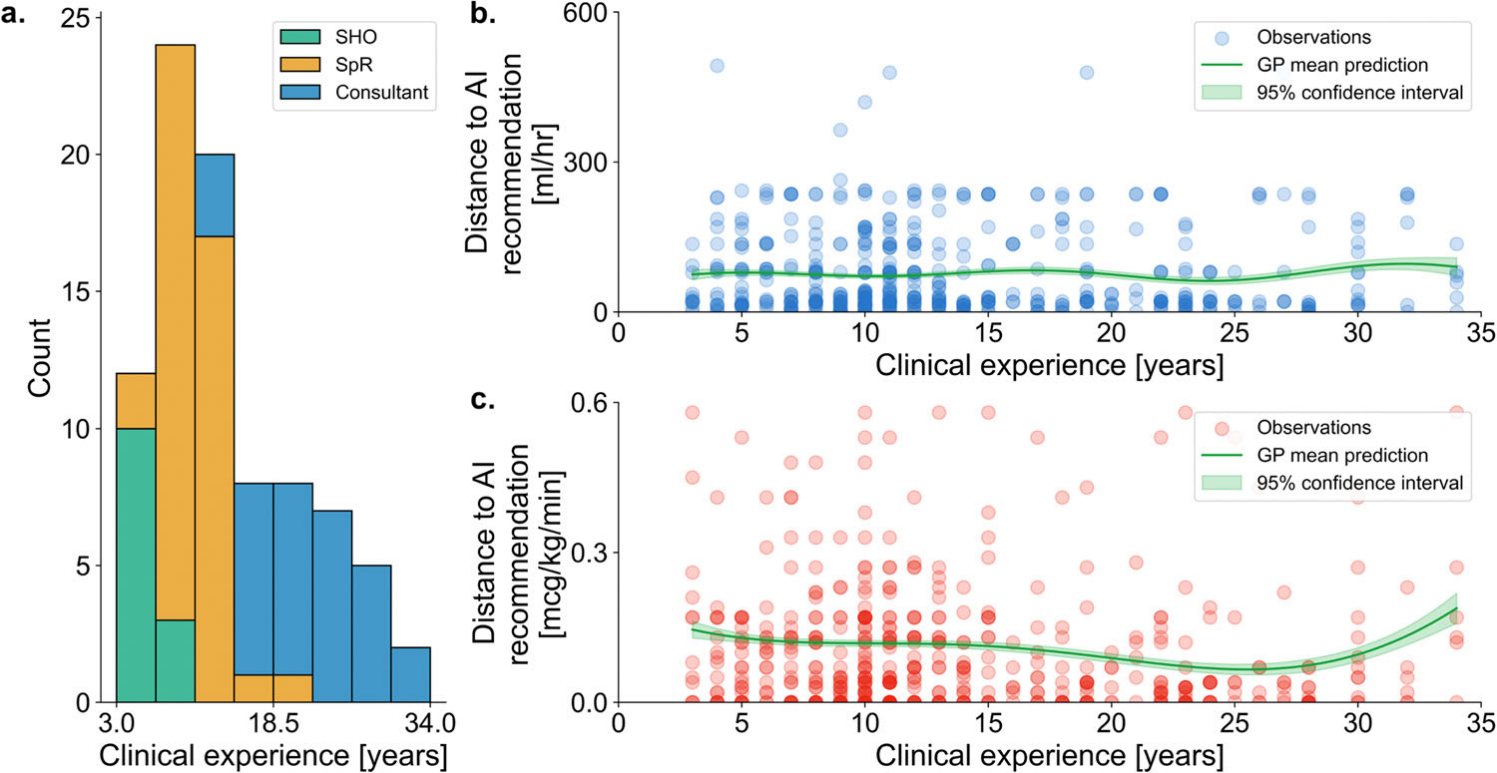
Impact of duration of clinical experience on adherence to AI suggestions. The distribution of experience levels among the three categories of seniority is shown in (**a**) (Consultant, most senior and equivalent to attending in the United States (US); SpR, specialist registrar and equivalent to fellow in the US; SHO, senior house officer and equivalent to resident in the US). Experience level was compared to AI adherence (in the form of absolute difference from the AI suggested dose) for both fluid (**b**, blue) and vasopressor (**c**, red). Lower value indicates higher adherence. Dot transparency in (**b**) and (**c**) represents density of points at any given location. ‘GP mean’ refers to a predicted Gaussian Process regression fit of the data with accompanying 95% confidence interval.

Clinician attitude to AI was extracted as a principal component of the four pre-experiment AI enthusiasm questions subjects were asked (Fig. 2a). The first component explained 69% of the variance (Fig. 2b). Attitude to AI did not have a significant linear association to the difference between subject selected dose and AI recommended dose for either fluid (*r* = −0.078, *p* = 0.075 by LLSR (linear least-squares regression)) or vasopressor (*r* = −0.074, *p* = 0.092 by LLSR), see Fig. 2c. Similarly, years of clinical experience did not have a significant association to the difference between subject selected dose and AI recommended dose for either fluid (*r* = 0.001, *p* = 0.862 by LLSR) or vasopressor (*r* = −0.086, *p* = 0.047 by LLSR), see Fig. 3b, c. Practice variation and adherence to AI by grade of doctor are shown in Supplementary Fig. 2.

### Clinician opinions on AI and the explanations

**Fig. 4:**
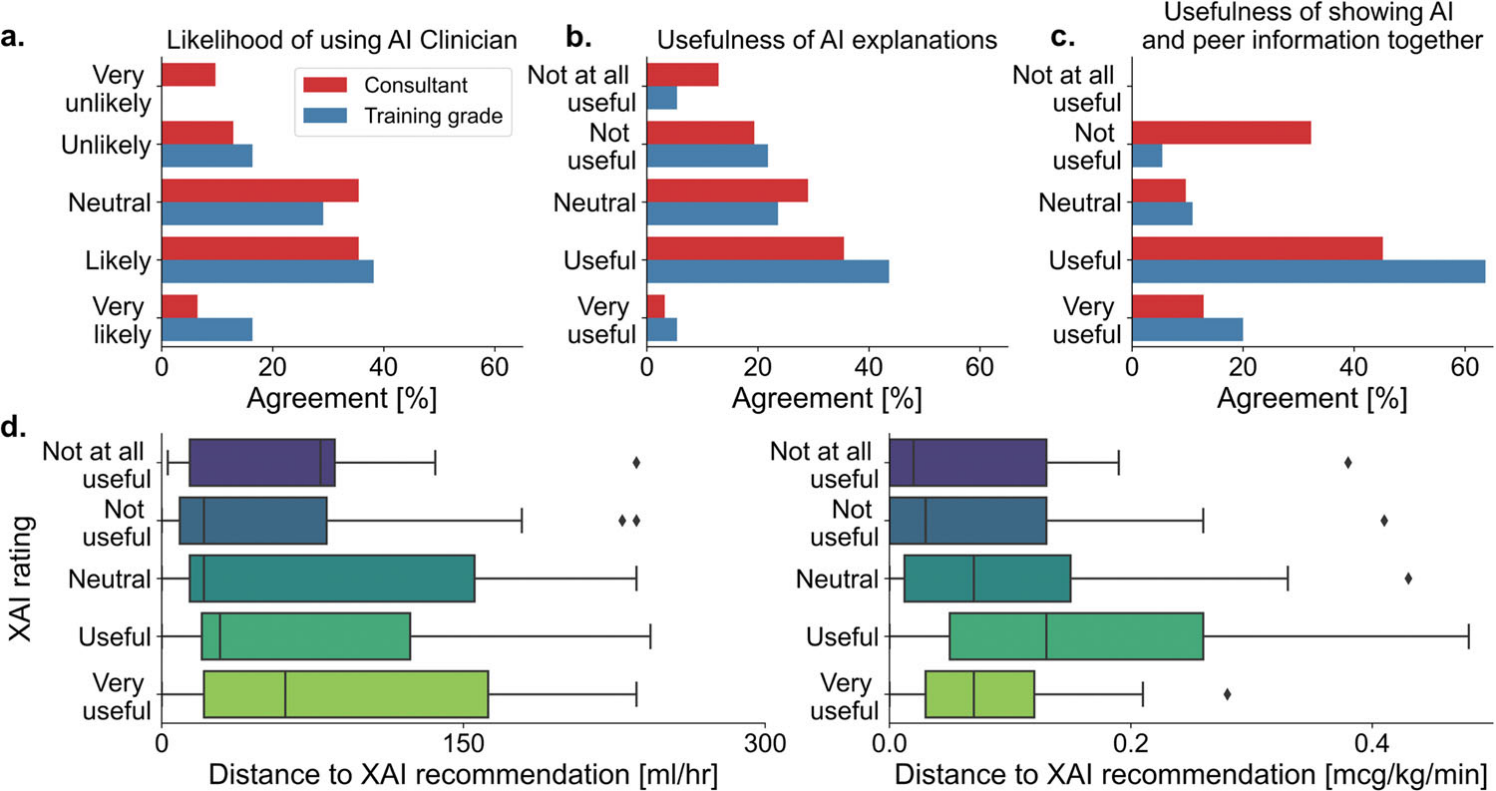
Post-experiment questions. **a**–**c** Shows responses to post-experiment questions broken down by training status. Self-reported usefulness of explanations is also plotted against adherence to XAI recommendation for both fluid (**d**, left) and vasopressor (**d**, right). Lower values indicate greater adherence and vice versa. For each boxplot, the centre line represents the median, box edges represent upper and lower quartiles, whiskers represent 1.5× inter-quartile range and diamonds are outliers. Colour spectrum gradient over boxplots relates to how useful XAI was rated.

Post experiment, subject likelihood of using an AI system for sepsis prescriptions on a scale from 1 to 5 (higher more likely to use) was mean 2.55 for training doctors (which encompasses both junior and intermediate doctors, SD 0.96) versus 2.16 for non-training doctors (senior/consultants, SD 1.07), *p* = 0.091 by independent *T*-test (Fig. 4a). Subjects were asked to rate the usefulness of the explanations on a scale from 1 to 5 (higher more useful) with mean 2.22 for training doctors (SD 1.03) versus 1.97 for non-training doctors (SD 1.11), *p* = 0.296 by independent *T*-test (Fig. 4b). Self-reported usefulness of explanations did not correlate with adherence to XAI suggestions (Fig. 4d). Subjects were also asked to rate the usefulness of showing peer and AI suggestions together on a scale from 1 to 5 (higher more useful) with mean 2.98 for training doctors (SD 0.73) versus 2.39 for non-training doctors (SD 1.09), *p* = 0.003 by independent *T*-test (Fig. 4c). Finally, subjects were also asked to rate importance of evidence on a 1 to 5 scale (higher more important) for their use of an AI system with a mean rating of 3.01 (SD 0.85) for observational evidence versus mean 3.33 (SD 0.76) for randomised clinical trial evidence, *p* = 0.011 by independent *T*-test.

## Discussion

This study has several important findings that add to our understanding of how prescription decisions can be influenced by AI-driven decision support recommendations. First, additional information (peer, AI or XAI) has a strong influence (proxied by dose shift) on prescription decisions (significantly for AI, not so for peers). However, whether the recommendation came in a plain form (AI alone) or garnished with an explanation (XAI, here simple feature importance) did not make a substantial difference. Second, inter-clinician dose variability was differentially affected according to whether the recommendation (whether peer, AI or XAI) was higher or lower than what subjects in the baseline arm did and this suggests that decision support systems might have a mixed impact on practice variation when deployed in a live setting. Third, there was no correlation between attitudes to AI or clinical experience on the AI-supported decisions suggesting a certain unvarying degree of AI acceptance in clinical experts, or one moderated more by variability in patient scenario than the clinician themselves. Fourth, there was no correlation between what doctors self-reported about how useful they found the XAI and whether the XAI actually influenced their decisions which brings into question the reliability of using XAI self-reports as an outcome metric in clinical XAI studies.

These findings should be considered in the context of several limitations. First, the rendition of the XAI condition used feature importance. While this is a commonly used XAI modality (both within and outside of healthcare)^12,19–23^, it is also on the lower end of the spectrum regarding what makes for a comprehensive explanation according to cognitive science^24^. Indeed, the variability in modality of XAI can have different impacts on medical decisions depending on the complexity or ambiguity of the context as well as the user’s experience of an AI system^25,26^.

Second, while steps were taken to ensure a large and representative range of patient scenarios, this may still leave gaps in the patient state space. We intentionally developed a two-pronged selection strategy to ensure our scenario choices were as generalisable as possible (see Supplementary Methods 1 which suggests reasonable coverage of the MIMIC state space presented graphically as a function of three principal components for visual purposes). Third, the patient scenarios were low fidelity owing to the experimental vignette format. Whilst this allows for a high degree of standardisation of scenarios, the dynamic nature of evaluating a real patient over time and learning what effect a given treatment has (or does not have) is therefore missing. Fourth, although our sample of doctors was large compared to similar clinical studies, it nevertheless relied on convenience sampling and therefore may not be representative of the medical population as a whole. Fifth, the AI suggestion itself was an isolated message containing the recommended doses for fluid and vasopressor without any confidence bounds or ranges. It may be that adherence to AI suggestions would be higher when presented with estimates of certainty, when presented graphically or using other algorithmic approaches^27^. Sixth, we do not have a detailed insight into the decision-making process that physicians internally performed. All we have observed in this study is self-reported data (which can be biased in many directions and from many causes) and final prescription data. Therefore, our conclusions are based on inferring that the input conditions (baseline, peer, AI, XAI) had a direct impact on the final prescription decisions via some internal cognitive effect on decision-making. Alternate methodologies to further explore this decision-making process might involve qualitative interviews and ‘think-aloud’ studies in which the verbalisation of decision-making is encouraged and formally analysed at aggregate level using thematic analysis. As a result, our interpretation of the study findings and conclusions are based on a data-driven framework of values rather than a more cognitive theoretical framework about *how* exactly the decision-making is influenced.

Notwithstanding these limitations, reviewing our findings alongside existing literature provides important insights into how we can improve the design and deployment of AI-based medical decision support tools. Attempts to quantitatively evaluate AI recommendations in general (non-clinical) problems have demonstrated the sometimes counterintuitive nature of how explainability impacts on performance. In one experiment, the response shift (a marker for AI influence similar to our experiment) was greater when an explanation was provided^6^. However, the quality of the explanation (good vs. poor) did not affect this shift with the authors suggesting that subjects might have been reassured by the presence of an explanation when the AI performance itself was good rather than actually assessing explanation quality or fidelity^6^, a potential form of automation bias. In a clinical environment, the consequences of erroneously acting on poor advice can be considerable with a study among 50 general practitioners (GPs) demonstrating that only 10% were able to correctly disagree with incorrect AI advice on a dermatology problem^28^ while in a radiology setting even task experts were not immune to the effects of poor AI advice (although they were considerably better at rejecting it than non-task experts)^8^.

Explanations have traditionally been posited as a means of rescuing users from poor AI advice though this has not clearly been borne out in clinical studies. For example, in a study investigating a psychiatric medication decision support tool, the presence of explanations did not provide rescue from intentionally poor AI recommendations suggesting a level of automation bias that could be problematic in a real-world clinical environment^10^. In our study, the explanation did not significantly increase adherence above and beyond the AI suggestion with several possible causes. It could be that trust and adherence was maximally achieved in most subjects by the AI suggestion alone leading to a ceiling effect. Or the very basic nature of the XAI was not persuasive. Or perhaps the nature of needing to make repetitive and cognitively burdensome decisions led users to quickly adopt a heuristic, one way or the other, as to whether they used the explanation in their decision-making or not. Further still, there were several users who commented that the variables given by the AI as part of the feature importance explanation did not seem physiologically plausible (see Supplementary Note 1 for a selection of post-experiment subject comments). This poses a paradox to those designing XAI systems. On the one hand, some might argue that a more complex explanation could satisfy users and lead to higher adherence. However, the strength of AI in being able to identify patterns in large datasets that are imperceptible to human clinicians can also be a weakness with regards to developing XAI. If some clinicians associate the quality of an AI explanation with physiological plausibility, then is an AI explanation based on patterns that human clinicians don’t usually see in their practice likely to be persuasive? Probably not. Ultimately, a mixed qualitative-quantitative approach might be critical for getting a better insight into the decision-making process that physicians use and where AI can be of most benefit and least harm during that process.

Other pertinent topics within medical AI-driven decision support research include the purported source of the advice as well as the experience level of the clinical audience receiving it. Gaube and colleagues studied both clinical experts and non-task experts for a chest radiograph diagnostic challenge with XAI suggestions. They found that experts rated their confidence as higher when advice was labelled as originating from an AI (although their accuracy was unchanged) while non-task experts had improved accuracy with the provision of explanations (compared to experts who did not)^9^. The peer suggestions in our study are not directly comparable as they were genuine rather than synthetic suggestions (and so differed in magnitude from the AI recommendations). However, we nonetheless also found that AI suggestions were more influencing than peers as a source of suggested advice. We did not, though, find any association with clinical experience level on adherence to AI.

Taken together, our findings on a comparatively large clinical expert population raise important questions for the meaning and design of medical XAI systems. Specifically, we show that the marginal impact of XAI was low in this experimental setting. The exact type, presentation and feedback loops for medical XAI systems that actually influence doctors remains unclear. It seems very likely that future research will need to more comprehensively consider social and cognitive aspects of decision-making alongside technical deployment of AI systems. We also cast doubt on the utility of self-reports as a valid metric for assessing XAI in clinical experts. Further work in this area could look to higher fidelity and more granular markers that assess the natural behaviour of clinicians when they interact with decision support tools. Answering these questions will be critical for bridging the translation gap between theoretical medical AI and real-world bedside implementation.

## Methods

### Data source and AI clinical decision support system

The ‘AI Clinician’ is a reinforcement-learning based intensive care unit (ICU) clinical decision support system that provides semi-autonomous continuous dosing suggestions for intravenous (IV) fluid and vasopressors^12^. The ‘AI Clinician’ was trained on the data of 17,083 ICU patients from the MIMIC-III database as previously described^12^. MIMIC-III is an anonymised, open-access database of over 60,000 ICU admissions from 2001–2012 in six teaching hospital ICUs from Boston in the United States^29^. Briefly, patients selected for training by the AI Clinician were adults with sepsis as defined by the sepsis-3 criteria^30^. Each patient’s data were split into 4-hour time blocks. For every 4-hour time block for each of the 17,083 patients, the AI Clinician clustered the patient into one of 750 states and produced a suggested dose for intravenous fluid and noradrenaline (the most commonly used vasopressor agent in septic shock)^31^.

Twenty four patient scenarios were chosen for inclusion in the experiment. Twelve of these were ‘expert selected’ by trying to ensure representation from four broad categories: (i) three patients where both the fluid and vasopressor AI dose suggestions were similar to what human clinicians had done in MIMIC-III, (ii) three patients where only the AI vasopressor suggestion was similar to humans (iii) three patients where only the AI fluid suggestion was similar to humans and (iv) three patients where neither AI fluid nor vasopressor suggestions were similar to humans. These 12 patients also spanned scenarios where the patient was receiving anywhere from no vasopressor to a large dose (>0.5 mcg/kg/min of noradrenaline-equivalent), again to ensure a representative patient mix. The other 12 patients were chosen by clustering the entire MIMIC-III sepsis dataset of 17,083 patients into 12 clusters and then selecting a patient within the closest percentile to the cluster centroid. This resulted in 12 patients that were less sick (as defined by proportion on vasopressor support and APACHE score) than the initial 12 but that were more representative of the MIMIC-III septic cohort. The amount of fluid and vasopressor support is shown in Supplementary Methods 1, separated by whether the patient was ‘expert-selected’ or ‘cluster-derived’.

### Vignette experiment and conditions

We conducted an experimental human-AI interaction vignette study for doctors using a modified between-subjects design. There were four experimental arms. In every arm, subjects were provided with patient data in the form of a fixed variables table (e.g. age, gender, weight), an interactive graph displaying a limited set of time varying features and a second larger table showing all time varying features (see Supplementary Methods 2 for screenshots). This was designed to look similar to the way in which most ICU doctors in the UK encounter patient data on their respective electronic health records (EHRs).

**Fig. 5:**
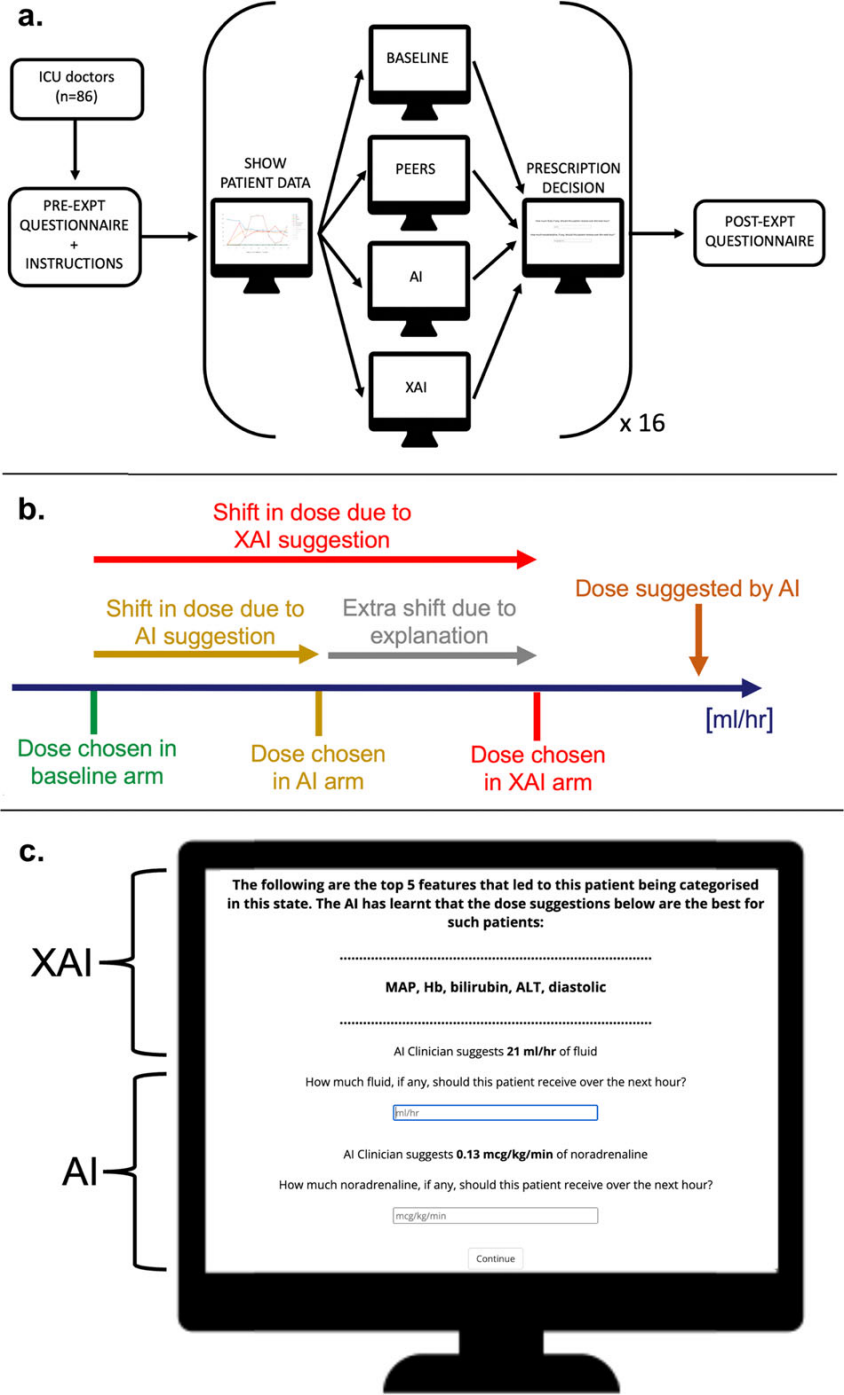
Dose shift and experiment protocol. The experiment protocol is shown in (**a**). Dose shift relative to baseline occurring as a result of showing the AI suggestion is shown in (**b**). The extra shift between AI and XAI is the marginal shift attributable to the explanation. **c** Shows the AI and XAI components of the decision screen where participants input their prescription choice.

Each subject (ICU doctor) performed 16 trials (Fig. 5a). The first four trials were identical for all subjects and were used as a pre-training period. The subsequent 12 trials comprised the main experiment. For each trial, subjects were asked to select a dose for fluid and a dose for vasopressor to be applied for the next hour. We used a multi-factorial experimental design with four arms, where each clinician experienced all four arms on different subsets of our 24 patients. The four arms were: baseline with no additional AI or peer human information (baseline); additional peer human clinical information (peer, see description below); additional AI decision support system information (AI); additional AI decision support system with explanation of the AI decision (feature importance, XAI). Examples of all four scenarios are available in the Supplementary Methods 2.

For the baseline scenario, subjects viewed only the patient data. For the peer human clinician scenario, subjects were also shown the probability density function of IV fluid and vasopressor doses prescribed by other doctors in the MIMIC-III dataset for patients in the same state. This can be thought of as a proxy for what peer clinicians have previously done for similar patients. This data was displayed as a violin plot (consisting of a conventional box plot with an overlaid distribution for the data derived via a kernel density estimation (KDE)). The rationale for including peer data as an experiment arm was to evaluate if clinicians merely want additional information or context to support their decision (regardless of source) or whether there is something specific about an AI suggestion that is more or less persuasive than simply knowing what their peers typically do.

For the AI scenario, subjects were also shown the AI Clinician suggested doses for fluid and vasopressor in text form. For the XAI scenario, subjects were shown the AI Clinician suggested doses as well as an explanation based on feature importance. The state space for the AI Clinician was constructed using a k-means clustering algorithm. After the algorithm converged, the cluster centroids represented the average feature values for patients in a particular state/cluster. A new patient would be assigned to the state/cluster that minimised the distance from their feature values to the respective cluster centroid. Intuitively, with over 40 features, some features will be closer to the cluster centroid value than others for any patient assigned to a given state. This is exploited to rank features in terms of their proximity to the cluster centroid (or average state feature values) given that the archetypal patient for whom an RL agent policy action most applies is a patient who is most typical of that state. Subjects were shown the top five ranked features contributing to state assignment (for details see Supplementary Methods 3). Although feature importance can be considered a basic form of XAI, it is nonetheless in widespread use within medical studies^12,19–23^.

The trial design matrix (see Supplementary Methods 4) ensured that half the subjects saw a patient under one arm while the others encountered the same patient under a different arm, allowing estimation of between arm variability by controlling for the patient. Our primary measure of interest was the difference in prescribed dose to the same patient across the four different arms—effectively measuring the shift in dose across arms as a measure of impact that the arm has on clinical decisions (Fig. 5b). The overall order of trials was varied to counterbalance any learning effects. Statistical analyses included two-sided *T*-tests for comparison of means (after confirming normality) and linear regression for assessing associations. Both were performed with no adjustment for multiple comparisons.

### Subject recruitment and experiment conduct

The experiment was created as an interactive web page using HTML and JavaScript (jsPsych library) that could run locally on a laptop. Pre-cleaned data from MIMIC-III patients trained on by the AI Clinician were checked for consistency and then feature values were converted to standard clinical UK units.

Clinician demographics, experience and affinity to AI were collected using a questionnaire prior to completion of the main experiment (Fig. 5a). After the experiment, subjects further completed a short post-experiment questionnaire (see Supplementary Methods 5). Data collected for each patient scenario included: clinician’s prescription doses for fluid and vasopressor per patient scenario as well as time taken per patient scenario.

A convenience sample of ICU doctors was recruited with the following inclusion criteria: (i) practising doctor, (ii) has worked for at least 4 months in an adult ICU, (iii) currently works in ICU or has worked in ICU within the last 6 months. Participants had the opportunity to participate remotely via Zoom or in person. Electronically recorded informed consent was obtained from all participants and each experiment lasted approximately 45 minutes. The study was approved by the Research Governance and Integrity Team (RGIT) at Imperial College London (ICREC reference 21IC7245). The institutional review board of the Massachusetts Institute of Technology (no. 0403000206) and Beth Israel Deaconess Medical Center (2001-P-001699/14) approved the use of MIMIC-III for research. Because our study made use of fully anonymised patient data, individual patient consent was not required.

### Reporting summary

Further information on research design is available in the Nature Research Reporting Summary linked to this article.

### Supplementary information

Supplemental Information

Reporting Summary

## Data Availability

The data (in CSV format) that support the findings of this study are available online at: 10.6084/m9.figshare.23192624.

## Code availability

The code (in the form of Jupyter notebooks) that support the findings of this study are available online at: 10.6084/m9.figshare.23192624.
