# Supplementary material for: Quantifying the impact of AI recommendations with explanations on prescription decision making
Source: NPJ Digit Med. 2023 Nov 7;6:206. doi: 10.1038/s41746-023-00955-z (PMC10630476; doi:10.1038/s41746-023-00955-z)
Supplement: Supplementary file 1 — Supplemental Information [file 41746_2023_955_MOESM1_ESM.pdf]

Supplementary Figure 1: Dose shift by condition for all 24 patient scenarios

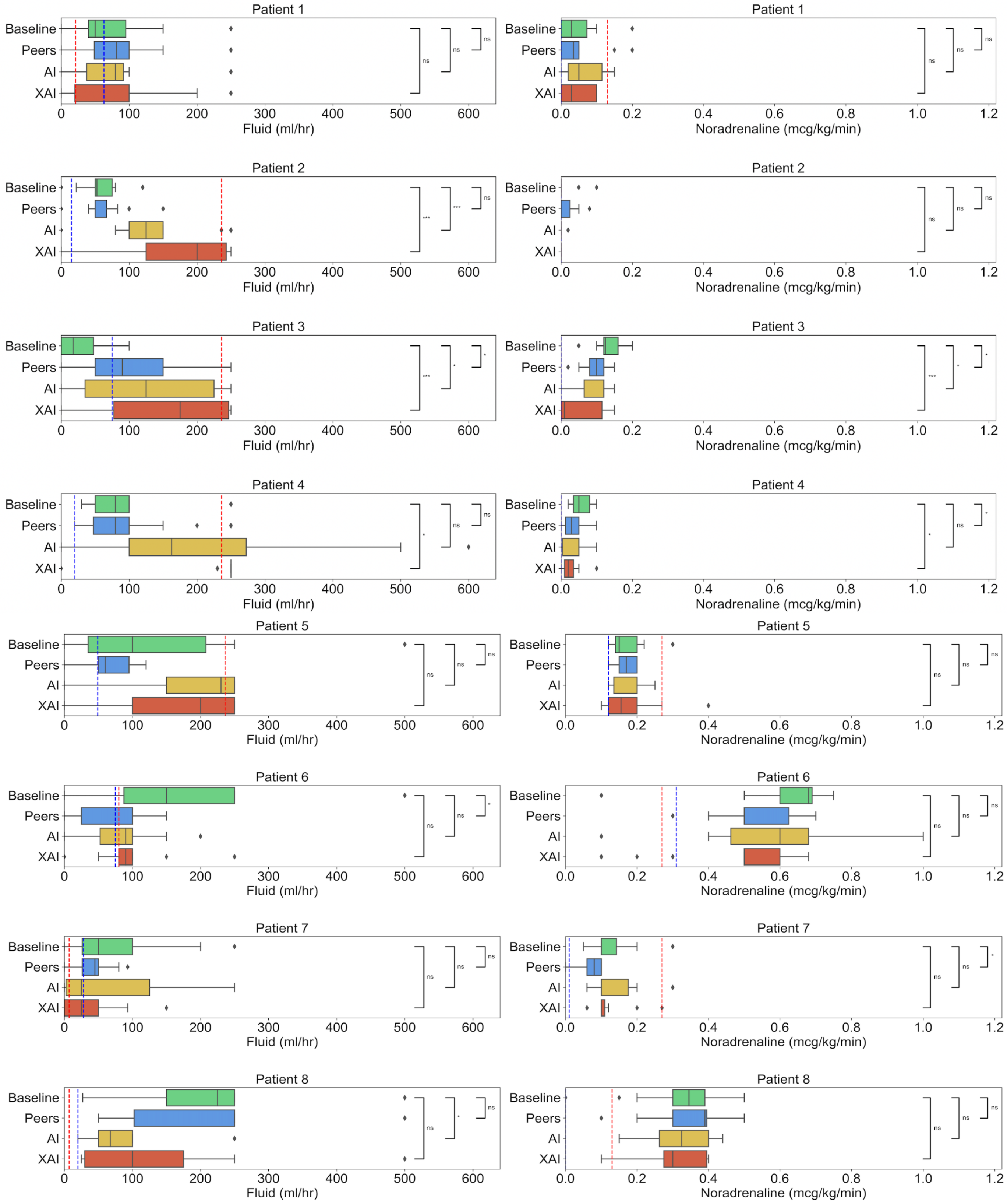

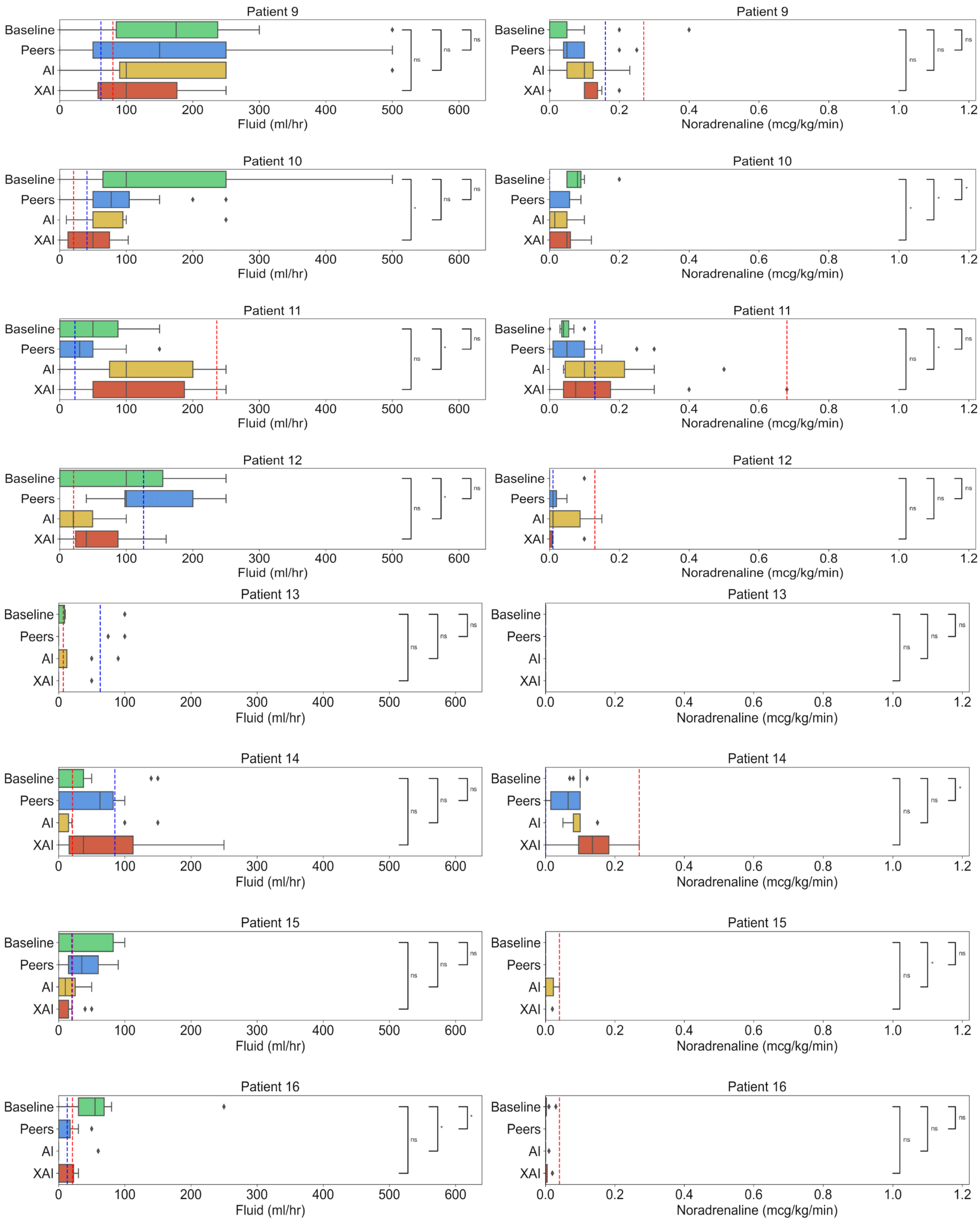

Patient 9
Patient 10
Patient 11
Patient 12
Patient 13
Patient 14
Patient 15
Patient 16
Baseline
Peers
AI
XAI
Fluid (ml/hr)
Noradrenaline (mcg/kg/min)
0
100
200
300
400
500
600
0.0
0.2
0.4
0.6
0.8
1.0
1.2
ns
*

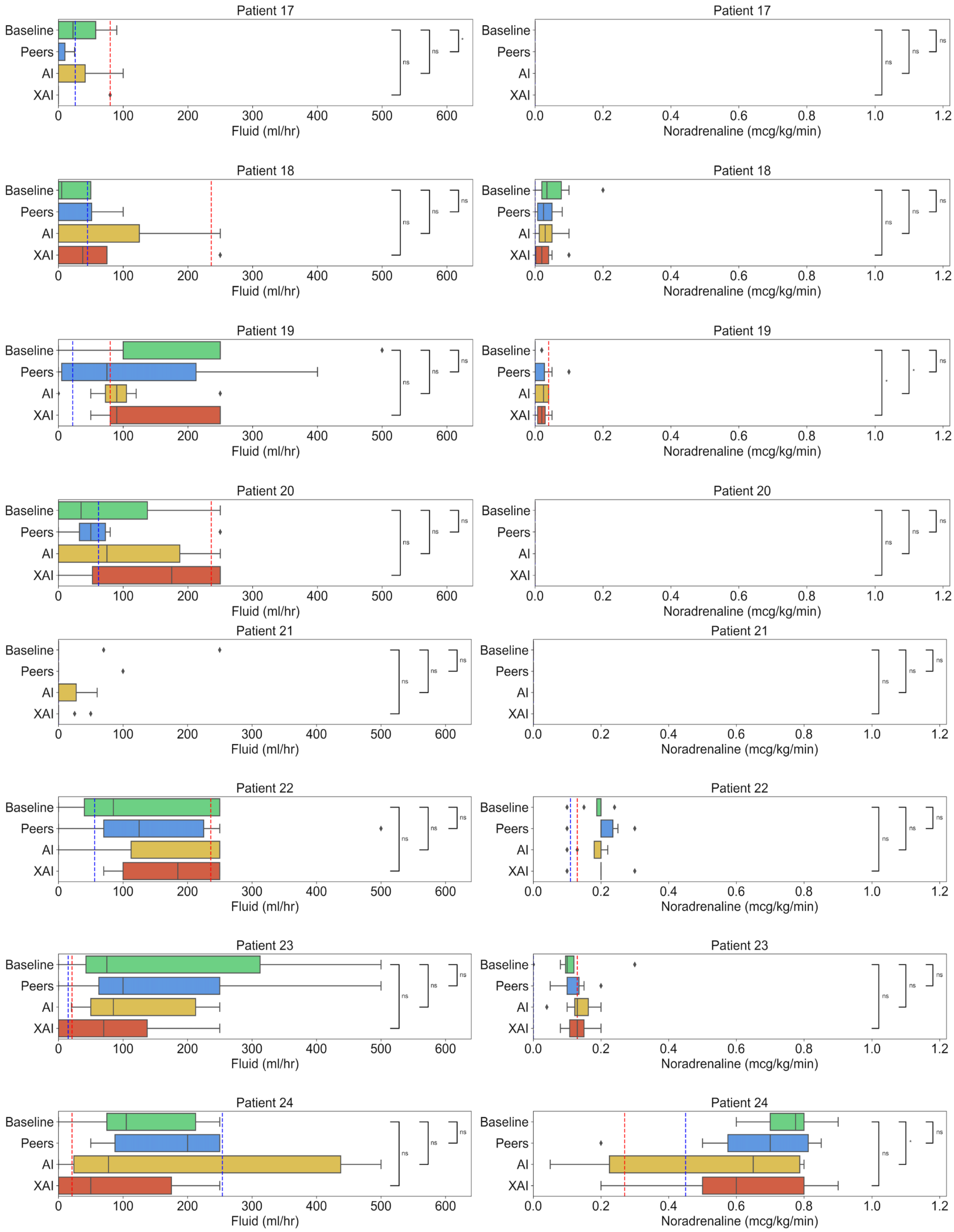

Supplementary Figure 1 legend. Boxplots of prescription dose distributions by trial arm (different box colour for each arm, same colour scheme as Figure 2 in manuscript) for either fluid or vasopressor and for each patient scenario. For each boxplot, the centre line represents the median, box edges represent upper and

lower quartiles, whiskers represent 1.5x inter-quartile range and diamonds are outliers. Blue dashed line represents the median of the peer distribution data (only available to those in the 'Peers' arm). Red dashed line represents the AI suggested dose (only available to those in the 'AI' or 'XAI' arms). Significance stars are obtained from independent T-tests for the means of the distribution ('Baseline' vs. intervention arm) with 'ns' referring to non-significant, a single star referring to a p-value <0.05 and three stars referring to a Bonferroni corrected p-value <0.000694 (for 72 comparisons in this figure).

Supplementary Figure 2: Data on practice variation and adherence to XAI

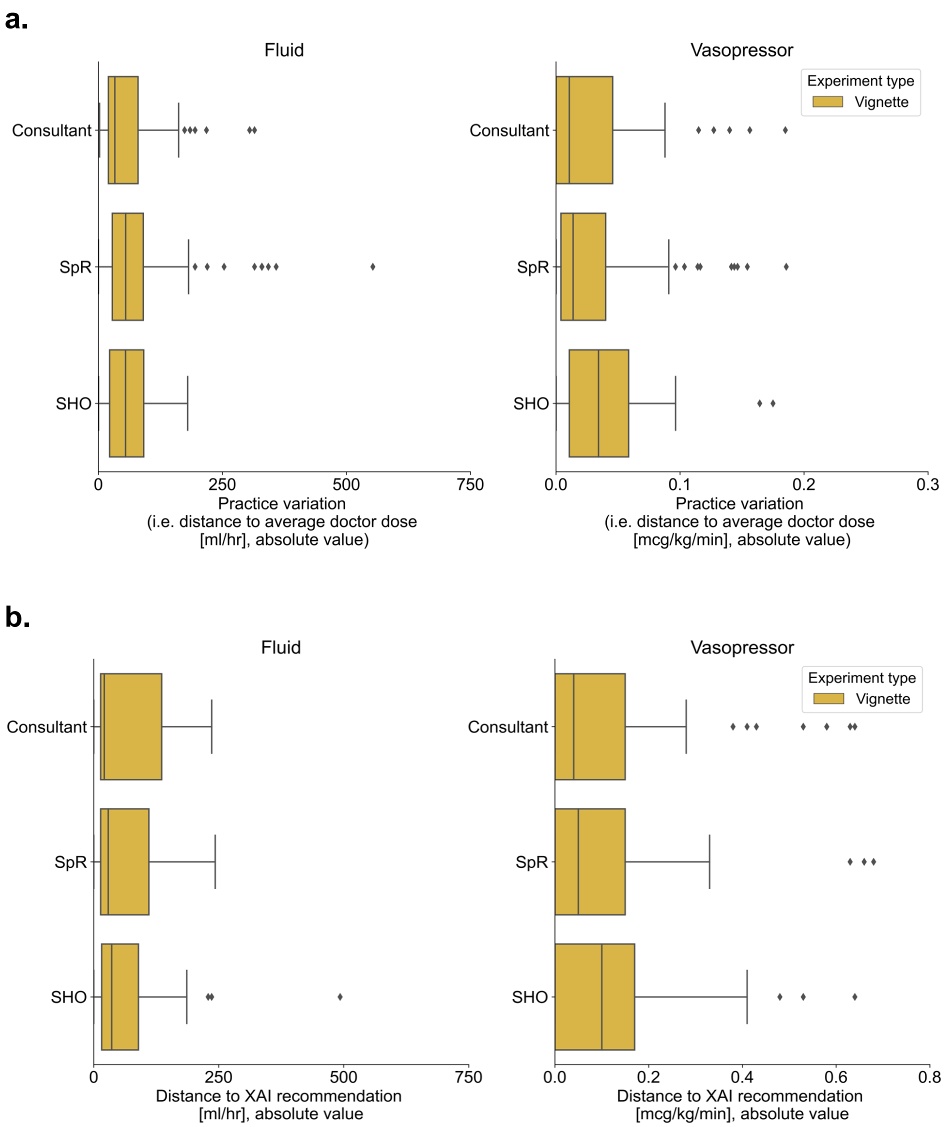

Supplementary Figure 2 legend. Boxplots on (a) practice variation or (b) adherence to AI by position/experience (Consultant, most senior and equivalent to attending in the United States (US); SpR, specialist registrar and equivalent to fellow in the US; SHO, senior house officer and equivalent to resident in the US). For each boxplot, the centre line represents the median, box edges represent upper and lower quartiles, whiskers represent 1.5x inter-quartile range and diamonds are outliers.

Supplementary Methods 1: Selection of patient scenarios - cluster derived vs. expert picked

As described in the methods, 24 patient scenarios were chosen for inclusion in the experiment. 12 were expert selected to reflect a range of fluid and vasopressors doses (blue colour). The remaining 12 were selected by clustering (k-means) the entire MIMIC database and selecting a patient near each of 12 cluster centroids (orange colour).

The patient scenarios are plotted below on a 3-component PCA model of MIMIC with dot size reflecting the dose of fluid (left figure) or vasopressor (right figure) (larger size = higher dose).

Overall, there is good coverage of the MIMIC state space.

FLUID

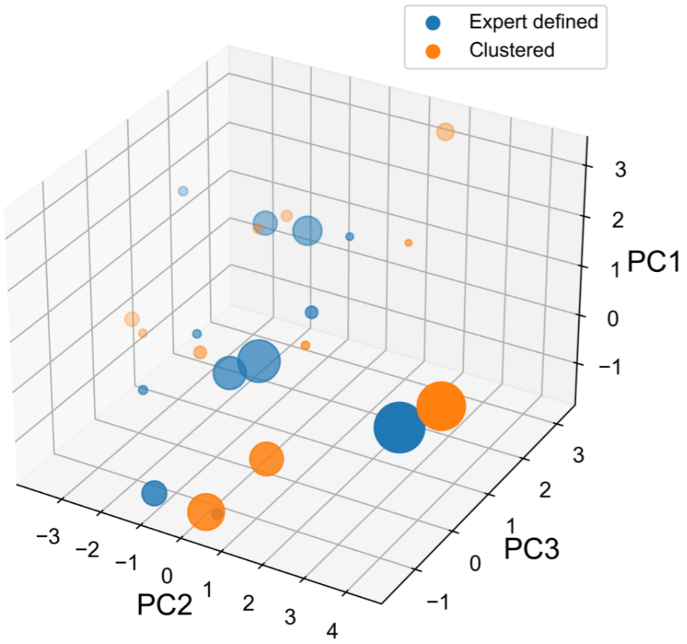

VASOPRESSOR

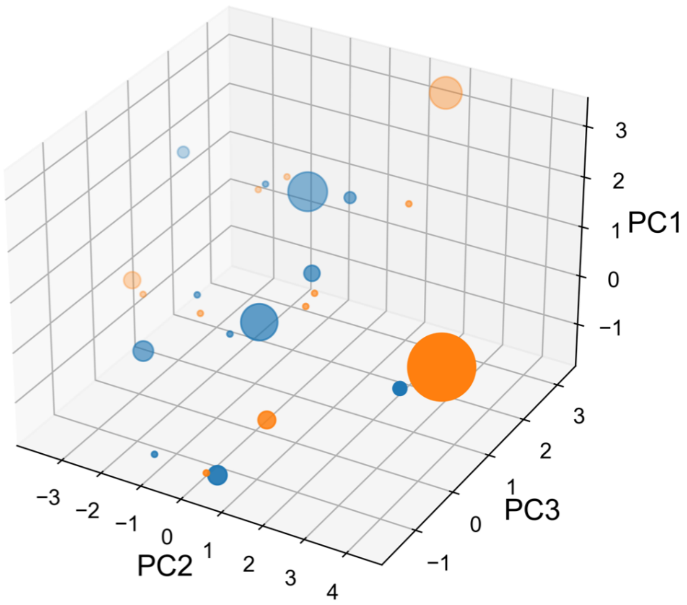

Supplementary Methods 2: Experiment screenshots

Completion Progress

Background and instructions

- Before starting the experiment, we will introduce the Artificial Intelligence (AI) Clinician, an AI decision support system for sepsis.
- The motivation for its development was to help with fluid and vasopressor decisions for patients in ICU with sepsis.
- Large amounts of electronic health data are now available.
- The volume of this data is larger than any human clinician might see in their entire career.
- If we can use computers to spot patterns in this data and generate insights, we might be able to better individualise our treatment decisions.

- The AI Clinician was trained on the data of 17,000 ICU patients and validated on a separate database of 85,000 ICU patients.
- It starts off by splitting the data for each patient into 4 hour blocks.
- It then categorises every 4 hour block for every patient into one of 750 states.
- These states can be thought of in the same say we might group patients in our head (e.g. elderly, overloaded, anuric, frail vs. young, fit, oliguric, dry) except that the computer does this on a much larger scale (750 different categories) using mathematical algorithms.
- The AI Clinician then watches the patient trajectories over time and learns, for any given state, which treatment doses were best for transitioning patients to healthier states and thus to increase their chance of eventual survival at 90 days.

< Previous Page 1/7 Next >

Completion Progress

In this experiment, we are interested in how ICU doctors interact with an AI decision support system for sepsis. You will be asked to review data for ICU patients with sepsis and asked what dose of fluid and noradrenaline, if any, you would give.

There are four different types of patient scenario that you will encounter and you will have access to patient data in all of them:

- **Baseline:** No AI information
- **Peers:** No AI information but you are shown what other clinicians did for similar patients
- **AI:** You get an AI suggestion for doses
- **AI with explanation:** You get an AI suggestion for doses and you are told which 5 features of the patient's data most contributed to them being assigned the state they are in. The AI has learnt for that state that its dose suggestions are optimal.

The type of scenario you are seeing will be flagged at the top of the page.

< Previous Page 2/7 Next >

Completion Progress

How much fluid, if any, should this patient receive over the next hour?

ml/hr

How much noradrenaline, if any, should this patient receive over the next hour?

mcg/kg/min

For the **baseline scenario**, there will be a response box like this for you to enter your dose selections after reviewing the patient data. The dose entry boxes will always be at the very bottom of every screen.

< Previous Page 3/7 Next >

Completion Progress

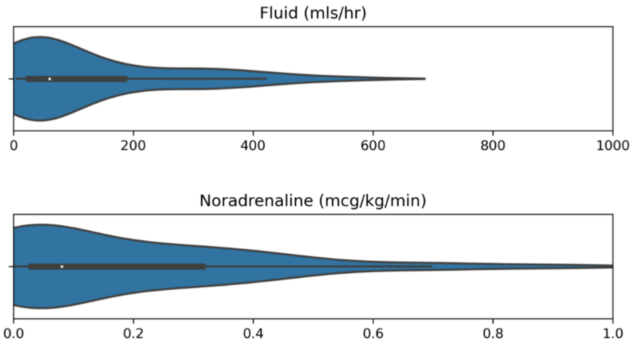

For the **peers scenario**, you will also be shown a figure like the one above which shows the distribution of human clinician actions for similar patients (those in the same state). As the databases are large, each of these figures shows the range of several hundred human decisions.

The white dot shows the median human action inside a boxplot. The blue area shows the distribution of human actions. The size of the blue area is largest where doses were more commonly chosen by human clinicians.

< Previous Page 4/7 Next >

Completion Progress

AI Clinician suggests **63 ml/hr** of fluid

How much fluid, if any, should this patient receive over the next hour?

ml/hr

AI Clinician suggests **0.03 mcg/kg/min** of noradrenaline

How much noradrenaline, if any, should this patient receive over the next hour?

mcg/kg/min

For the **AI scenario**, you will be given the AI suggested doses above the entry boxes for your dose decisions.

< Previous Page 5/7 Next >

Completion Progress

The following are the top 5 features that led to this patient being categorised in this state. The AI has learnt that the dose suggestions are the best for such patients:

MAP, lactate, INR, pH, age

AI Clinician suggests **236 ml/hr** of fluid

How much fluid, if any, should this patient receive over the next hour?

ml/hr

AI Clinician suggests **0 mcg/kg/min** of noradrenaline

How much noradrenaline, if any, should this patient receive over the next hour?

mcg/kg/min

For the **AI with explanation scenario**, you will be given a list of the top 5 (out of more than 40 data features) that contributed the most to the patient being assigned to the state they are in at the time point in which you are asked to make your decision. The AI has learnt that its dose suggestions are optimal for patients in this state.

< Previous Page 6/7 Next >

Completion Progress

Before you begin the experiment, you will have the opportunity to see an example patient data screen. The interactive graph is initially set to show HR, MAP, lactate, urine output, and hourly rates of fluid and noradrenaline.

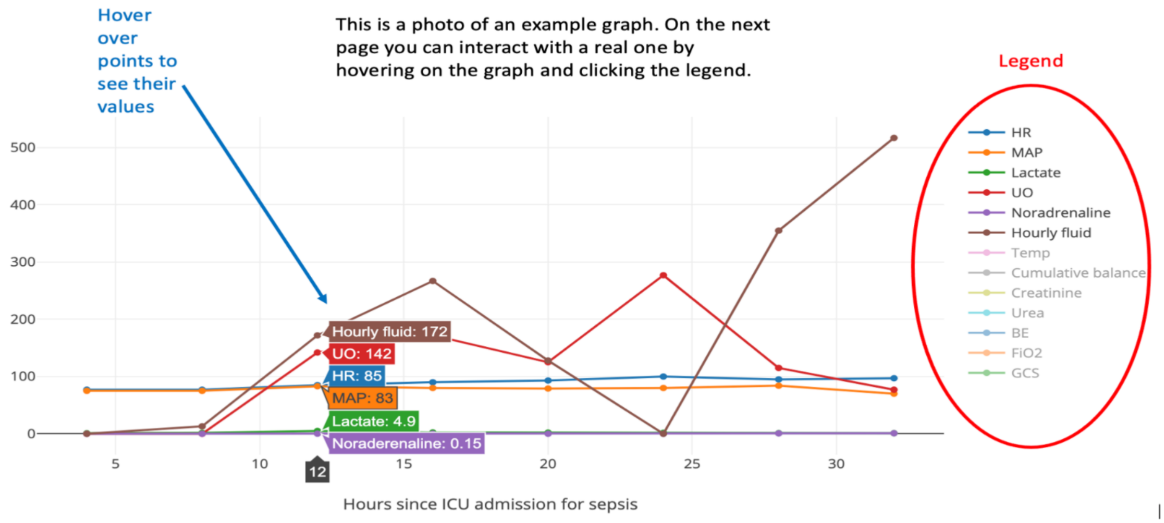

- **Hovering** over a point on the graph shows the values at that time point.
- A **single click** on a particular variable in the legend on the right hand side will show or hide that variable.
- A **double click** on a particular variable in the legend shows only that variable.
- A **further double click** will show all variables.

< Previous Page 7/7 Next >

Baseline scenario

Please scroll through the information below and make your dose selections at the bottom of the screen

Age	Gender	Weight (kg)	Admission SOFA	IPPV received
57	Male	99	4	Yes

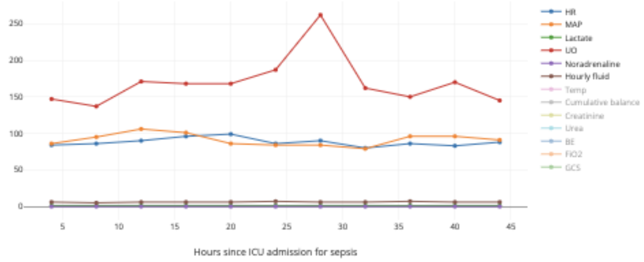

Hours since admission	4	8	12	16	20	24	28	32	36	40	44
HR	84	86	90	96	99	86	90	80	86	83	88
Systolic	141	150	167	158	144	140	146	132	150	150	149
MAP	86	95	106	101	86	84	84	79	96	96	91
Diastolic	66	72	79	76	65	62	62	61	74	74	70
Resp rate	27	29	27	17	16	17	28	25	23	25	26
SpO2	95	95	95	96	97	97	95	96	97	96	96
Temperature	38	38	37	36	37	36	37	38	37	38	38
GCS	10	10	11	11	11	11	11	10	10	11	11
FiO2	0.4	0.4	0.4	0.4	0.4	0.4	0.4	0.4	0.4	0.4	0.4
PF ratio	267	267	239	234	239	257	257	261	262	262	262
pH	7.47	7.47	7.44	7.44	7.44	7.46	7.46	7.44	7.44	7.44	7.44
PaO2	14.3	14.3	12.7	12.5	12.8	13.7	13.7	14	14	14	14
PaCO2	4.8	4.8	5.3	5.3	5.3	5.1	5.1	5.5	5.6	5.6	5.6
Base excess	2	2	2	2	2	2	2	2.8	3	3	3
Lactate	1.2	1.2	1.2	1.2	1.2	1.2	1.2	1.2	1.2	1.2	1.2
Bicarbonate	26	26	26	26	26	26	26	25	25	25	25
Glucose	9.5	10.4	10.1	8.4	8.4	7	6.7	10.1	9.8	7.9	8.8
Hb	12	12	12	12	12	12	12	12	11.8	11.5	11.5
White cell count	16.1	16.1	16.1	16.1	16.1	16.1	16.1	16.1	16.1	16.1	16.1
Platelet count	340	340	340	340	340	340	340	340	340	340	340
INR	1	1	1	1	1	1	1	1	1	1	1
PT	12	12	12	12	12	12	12	12	12	12	12
APTT	23	23	23	23	23	23	23	23	23	23	23
Sodium	141	141	141	141	141	141	141	141	141	141	141
Potassium	4.2	4.2	4.2	4.2	4.2	4.3	4.3	4.3	4.3	4.3	4.3
Urea	12.1	12.1	12.1	12.1	12.1	12.1	12.1	12.1	12.1	12.1	12.1
Creatinine	79	79	79	79	79	79	79	79	79	79	79
ALT	62	61	59	58	57	56	54	53	52	51	49
AST	40	40	39	38	38	37	37	36	35	35	34
Bilirubin	6	6	6	7	7	7	7	8	8	8	8
Albumin	21	21	21	21	21	21	21	21	21	21	21
Noradrenaline	0	0	0	0	0	0	0	0	0	0	0
Hourly input (ml)	6	5	6	6	6	7	6	6	7	6	6
Urine output (ml)	147	137	171	168	168	187	262	162	150	170	145
Cumulative input (ml)	14895	14915	14940	14965	14990	15020	15045	15070	15100	15125	15150
Cumulative output (ml)	18655	19205	19890	20565	21240	21990	23040	23690	24290	24970	25550
Cumulative balance (l)	-3.8	-4.3	-5	-5.6	-6.2	-7	-8	-8.6	-9.2	-9.8	-10.4

How much fluid, if any, should this patient receive over the next hour?

ml/hr

How much noradrenaline, if any, should this patient receive over the next hour?

mcg/kg/min

Continue

Peer human clinician scenario

Please scroll through the information below and make your dose selections at the bottom of the screen

Age	Gender	Weight (kg)	Admission SOFA	IPPV received
79	Female	52	8	Yes

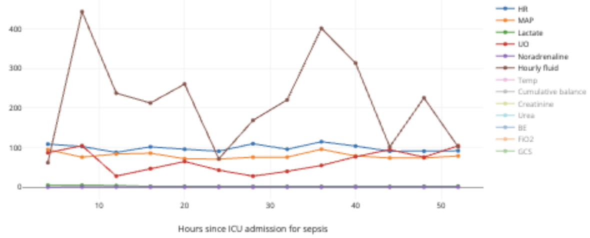

Hours since admission	4	8	12	16	20	24	28	32	36	40	44	48	52
HR	109	103	88	102	96	91	110	96	115	104	91	91	92
Systolic	137	95	95	113	97	93	99	104	124	114	116	115	122
MAP	95	76	84	86	72	71	76	76	96	79	74	74	79
Diastolic	64	52	45	53	45	42	51	50	60	64	57	56	60
Resp rate	17	17	14	12	12	12	12	13	14	13	12	12	13
SpO2	99	96	98	99	99	99	99	99	98	96	98	99	99
Temperature	36	36	36	37	37	38	38	38	39	37	37	37	38
GCS	6	6	7	7	8	8	8	8	7	8	6	6	6
FiO2	0.5	0.49	0.5	0.5	0.5	0.45	0.4	0.4	0.4	0.4	0.4	0.4	0.4
PF ratio	638	303	360	380	380	455	447	327	317	312	354	417	417
pH	7.35	7.32	7.33	7.33	7.33	7.36	7.36	7.35	7.33	7.32	7.32	7.33	7.33
PaO2	42.5	19.8	24	25.3	25.3	27.1	23.9	17.5	17	16.7	18.9	22.3	22.3
PaCO2	3.6	3.7	3.9	4.5	4.5	3.7	3.8	3.9	3.9	3.9	3.7	3.5	3.5
Base excess	-8.4	-9.7	-8.2	-6	-6	-7	-7	-7	-8.3	-9	-9.4	-10	-10
Lactate	4.8	4.8	4	1.8	1.8	1.3	1.3	1.3	1.3	1.3	1.3	1.9	2.5
Bicarbonate	13	13	15	16	17	15	15	16	15	15	14	14	14
Glucose	26.8	18.8	5.6	6	9.7	13.4	10.4	8.2	5.6	5.5	5.4	6.5	7.7
Hb	13.8	13.8	12.2	11.2	10.8	9.3	10.1	11.1	11.1	11	11.2	11.2	10.8
White cell count	21.3	21.3	21.3	19.3	17.8	17.8	16.8	15.9	15.9	15.9	15.9	15.9	14.8
Platelet count	297	297	351	353	341	341	289	245	223	185	185	185	175
INR	1.7	1.8	1.9	1.9	1.9	1.9	1.6	1.4	1.4	1.4	1.4	1.4	1.4
PT	16	16	17	17	17	17	15	14	14	14	14	14	14
APTT	150	147	96	66	32	43	42	39	39	39	39	39	39
Sodium	141	141	146	144	141	136	134	134	135	138	138	138	139
Potassium	3.8	4.7	3.8	4.3	5	4.7	5.1	5.5	5	4.1	4.1	4.1	4.2
Urea	7.5	7.5	7	6.4	6.1	5.8	5.7	5.7	5.6	5.4	5.4	5.4	5.4
Creatinine	106	106	99	87	79	86	97	106	102	97	97	97	92
ALT	190	190	190	190	190	190	190	190	190	190	190	190	190
AST	474	474	474	474	474	474	474	474	474	474	474	474	474
Bilirubin	5	5	5	5	5	5	5	5	5	5	5	5	5
Albumin	20	20	20	20	20	20	20	20	20	20	20	20	20
Noradrenaline	0.15	0.58	0.45	0.44	0.42	0.42	0.42	0.42	0.42	0.4	0.39	0.38	0.33
Hourly input (ml)	62	444	238	213	261	72	169	221	402	314	102	226	102
Urine output (ml)	87	105	28	47	65	43	28	40	55	77	95	76	105
Cumulative input (ml)	3450	5229	6182	7037	8082	8372	9050	9935	11543	12799	13207	14112	14522
Cumulative output (ml)	350	770	885	1074	1336	1508	1620	1782	2002	2310	2690	2995	3415
Cumulative balance (l)	3.1	4.5	5.3	6	6.7	6.9	7.4	8.2	9.5	10.5	10.5	11.1	11.1

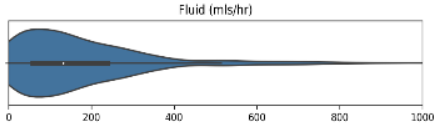

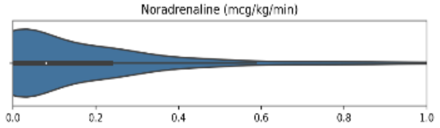

How much fluid, if any, should this patient receive over the next hour?

ml/hr

How much noradrenaline, if any, should this patient receive over the next hour?

mcg/kg/min

Continue

AI scenario

3 of 16

Please scroll through the information below and make your dose selections at the bottom of the screen

Age	Gender	Weight (kg)	Admission SOFA	IPPV received
67	Female	87	10	Yes

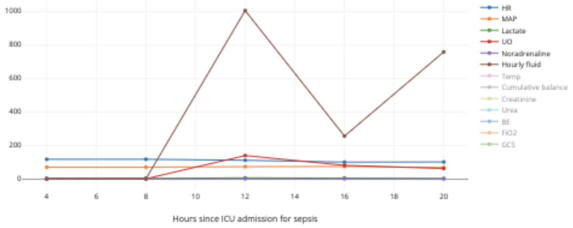

Hours since admission	4	8	12	16	20
HR	116	116	110	99	100
Systolic	92	92	102	107	100
MAP	69	69	72	73	67
Diastolic	58	58	61	57	52
Resp rate	14	14	14	14	23
SpO2	98	98	99	97	97
Temperature	36	36	36	36	36
GCS	3	3	5	8	4
FiO2	1	1	0.75	0.44	0.4
PF ratio	325	336	344	377	254
pH	7.46	7.43	7.38	7.48	7.48
PaO2	43.3	44.8	34.5	22	13.6
PaCO2	5.6	5.8	6	4.7	4.7
Base excess	6	4	0.7	3.6	2.7
Lactate	1.1	2	5.7	4.8	1.6
Bicarbonate	27	27	24	24	24
Glucose	10.4	9	7.6	7.2	7.4
Hb	11.8	8	11.6	10.8	10.6
White cell count	11.1	14.5	19.7	19.8	19.8
Platelet count	322	266	222	235	235
INR	1.3	1.3	1.6	1.6	1.6
PT	15	15	17	17	17
APTT	55	45	32	32	32
Sodium	139	137	136	137	137
Potassium	3.8	4.9	4.3	3.7	4
Urea	6.1	6.1	5.3	5	5
Creatinine	44	44	37	35	35
ALT	16	16	16	16	16
AST	23	23	23	23	23
Bilirubin	18	18	18	18	18
Albumin	33	33	33	33	33
Noradrenaline	0	0	0.17	0.17	0.17
Hourly input (ml)	0	0	1001	254	755
Urine output (ml)	0	0	138	80	61
Cumulative input (ml)	4025	4025	8031	9047	12069
Cumulative output (ml)	0	0	555	875	1120
Cumulative balance (l)	4	4	7.5	8.2	10.9

AI Clinician suggests **80 ml/hr** of fluid

How much fluid, if any, should this patient receive over the next hour?

[ml/hr]

AI Clinician suggests **0.13 mcg/kg/min** of noradrenaline

How much noradrenaline, if any, should this patient receive over the next hour?

[mcg/kg/min]

Continue

AI with explanation scenario

4 of 16

Please scroll through the information below and make your dose selections at the bottom of the screen

Age	Gender	Weight (kg)	Admission SOFA	IPPV received
72	Male	91	11	Yes

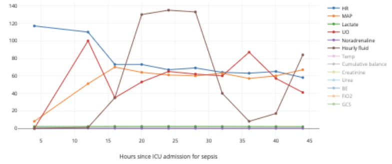

Hours since admission	4	12	16	20	24	28	32	36	40	44
HR	117	110	73	73	67	69	64	63	65	58
Systolic	8	83	114	101	100	99	101	94	101	120
MAP	8	51	70	64	61	60	63	57	60	67
Diastolic	8	41	57	55	51	47	51	45	47	49
Resp rate	36	34	26	30	22	25	26	21	22	19
SpO2	96	95	100	98	98	95	98	98	98	99
Temperature	36	36	36	36	36	36	36	36	36	36
GCS	11	11	10	10	10	10	10	10	10	10
FiO2	0.6	0.6	0.6	0.46	0.5	0.5	0.5	0.5	0.5	0.5
PF ratio	118	100	91	148	151	156	156	186	217	248
pH	7.44	7.44	7.44	7.37	7.36	7.39	7.39	7.37	7.36	7.34
PaO2	9.5	8	7.3	9	10.1	10.4	10.4	12.5	14.5	16.6
PaCO2	4.5	4.9	5.1	5.6	5.5	5.2	5.2	5.3	5.4	5.6
Base excess	0	0.7	1	-1.1	-1	0	0	-0.7	-1.3	-2
Lactate	2	2.1	2.2	2.2	2.2	2.1	2.1	2	2	1.9
Bicarbonate	25	25	25	25	24	24	24	20	20	20
Glucose	4.6	7.4	7.9	10.8	6.9	7.4	7.4	7.4	7.3	7.2
Hb	10.1	9.4	9.4	9.4	8.9	8.7	8.7	8.7	9.6	10.1
White cell count	18.8	16.8	16.8	16.8	16.5	15	15	15	15	15
Platelet count	352	305	305	305	313	354	354	354	354	354
INR	1.3	1.3	1.3	1.3	1.3	1.3	1.3	1.3	1.3	1.3
PT	15	15	15	15	15	15	15	15	15	15
APTT	36	36	36	36	36	36	36	36	36	36
Sodium	144	145	145	143	143	145	145	145	145	145
Potassium	3.9	4.3	4.3	4.7	4.7	4.6	4.6	4.6	4.6	4.6
Urea	11.4	11.4	11.4	11.8	11.8	11.8	11.8	11.8	11.8	11.8
Creatinine	88	97	97	106	106	106	106	106	106	106
ALT	24	24	23	23	23	22	22	21	21	20
AST	31	31	32	32	32	33	33	33	34	34
Bilirubin	9	10	10	10	10	10	11	11	11	11
Albumin	32	32	32	32	32	32	32	32	32	32
Noradrenaline	0	0.2	0.12	0.05	0.03	0.05	0.03	0.03	0.05	0.08
Hourly input (ml)	0	1	35	130	135	133	40	8	17	84
Urine output (ml)	0	100	35	53	65	62	60	87	57	41
Cumulative input (ml)	0	5	145	667	1209	1744	1906	1940	2009	2348
Cumulative output (ml)	0	400	540	755	1015	1265	1505	1855	2085	2250
Cumulative balance (l)	0	-0.4	-0.4	-0.1	0.2	0.5	0.4	0.1	-0.1	0.1

The following are the top 5 features that led to this patient being categorised in this state. The AI has learnt that the dose suggestions below are the best for such patients:

AST, INR, diastolic, SOFA score, ALT

AI Clinician suggests **7 ml/hr** of fluid

How much fluid, if any, should this patient receive over the next hour?

[ml/hr]

AI Clinician suggests **0.27 mcg/kg/min** of noradrenaline

How much noradrenaline, if any, should this patient receive over the next hour?

[mcg/kg/min]

Continue

Supplementary Methods 3: XAI feature importance

The approach leverages the fact that the state space for RL based sepsis algorithms is commonly constructed using a k-means clustering algorithm to enable dimensionality reduction. After the algorithm converges, the cluster centroids represent the average feature values for patients in a particular state/cluster.

A new patient would be assigned to the state/cluster that minimised the distance from their feature values to the respective cluster centroid. Intuitively, with often over 40 features, some features will be closer to the cluster centroid value than others for any patient assigned to a given state.

This is exploited to rank features in terms of their proximity to the cluster centroid (or average state feature values) given that the archetypal patient for whom an RL agent policy action most applies is a patient who is most typical of that state. So subjects can be shown the top five ranked features contributing to state assignment.

Supplementary Methods 4: Trial matrices

		REGION 1				REGION 2											
Subjects	Group	BA	PE	AI	XAI	BA	PE	AI	XAI	BA	PE	AI	XAI	BA	PE	AI	XAI
7	I	1	2	3	4	5	8	10	13	6	9	12	15	7	11	14	16
7	II	1	2	3	4	11	5	6	7	12	14	8	9	13	15	16	10
7	III	1	2	3	4	7	11	14	16	6	9	12	15	5	8	10	13
7	IV	1	2	3	4	13	15	16	10	12	14	8	9	11	5	6	7
6	V	1	2	3	4	10	7	5	14	9	6	15	12	8	16	13	11
6	VI	1	2	3	4	16	10	9	8	15	13	7	6	14	12	11	5
5	VII	1	2	3	4	8	16	13	11	9	6	15	12	10	7	5	14
5	VIII	1	2	3	4	14	12	11	5	15	13	7	6	16	10	9	8
Subjects	Group	BA	PE	AI	XAI	BA	PE	AI	XAI	BA	PE	AI	XAI	BA	PE	AI	XAI
5	IX	1	2	3	4	17	20	22	25	18	21	24	27	19	23	26	28
5	X	1	2	3	4	23	17	18	19	24	26	20	21	25	27	28	22
5	XI	1	2	3	4	19	23	26	28	18	21	24	27	17	20	22	25
5	XII	1	2	3	4	25	27	28	22	24	26	20	21	23	17	18	19
4	XIII	1	2	3	4	22	19	17	26	21	18	27	24	20	28	25	23
4	XIV	1	2	3	4	28	22	21	20	27	25	19	18	26	24	23	17
4	XV	1	2	3	4	20	28	25	23	21	18	27	24	22	19	17	26
4	XVI	1	2	3	4	26	24	23	17	27	25	19	18	28	22	21	20

The numbers from 1 to 28 refer to patient scenarios. Patient scenarios 1 to 4 (region 1) were common to all participants and did not form part of the primary analysis. Region 2 was designed so that half the subjects saw a patient under one arm while the others encountered the same patient under a different arm, allowing estimation of between arm variability by controlling for the patient. The study was not randomised.

Supplementary Methods 5: Pre- and post-experiment questionnaires

Pre-experiment:

- How old are you?
- For how many years have you been working clinically?
- What is your current position (or equivalent level)?
    - Options: ('SHO', 'SpR', 'Consultant')
- What type of ICU do you predominantly work in?
    - Options: ('General', 'Cardiac', 'Neuro', 'Paeds', 'Liver', 'Trauma', 'Mixed')
- Your opinions on Artificial Intelligence (AI) on a 5-point Likert scale ('Strongly disagree', 'Disagree', 'Neutral', 'Agree', 'Strongly agree')
    - AI will benefit society at large
    - AI will personally benefit me in my day to day life
    - AI will benefit the National Health Service (NHS
    - AI will personally benefit my work as a clinician

Post-experiment:

- How likely would you be to use an AI system like this to help guide your clinical practice in the ICU? Keep in mind that the current interface is a demo and will be replaced by a professionally designed version similar to current electronic health software.
    - 5-point Likert scale ('Very Unlikely', 'Unlikely', 'Neutral', 'Likely', 'Very likely')

- How important to you using an AI system like this is evidence of improved clinical outcomes (e.g. reduced length of stay or lower mortality) in an observational study?
    - 5-point Likert scale ('Very unimportant', 'Unimportant', 'Neutral', 'Important', 'Very important')

- How important to you using an AI system like this is evidence of improved clinical outcomes (e.g. reduced length of stay or lower mortality) in a randomised trial?
    - 5-point Likert scale ('Very unimportant', 'Unimportant', 'Neutral', 'Important', 'Very important')

- How useful overall were the explanations for the AI suggestions?
    - 5-point Likert scale ('Not at all useful', 'Not useful', 'Neutral', 'Useful', 'Very useful')

Supplementary Note 1: Selected comments from subjects

- I feel that the AI does help narrow my range of options to make a more evidence-based decision.
- At times I found I agreed with the AI system. At time I found I wildly diverged from it. I think mostly my responses were more extreme ('stop fluid', 'double norad') than the AI's suggestions. I really enjoyed this exercise.
- Different way of approaching decision making than I might normally do, obviously with less inputs than normally available. Prompts interesting thinking about how we make clinical decisions "ahead of time" for patients i.e. in the next hour, rather than being reactive. It would be really interesting to estimate how much effect the tool has on moderating my normal decision making.
- Very interesting. I thought the AI explanations would make me trust it, but it's difficult if you don't understand the algorithm / biological mechanism - I guess I expected it to use cardiac output parameters / SIRS criteria etc..
- Great tool - innovative and barring a few anomalies, feels pretty reflective of what I would do.
- The AI suggestions with explanations were not very well explained. 'Words' were given without context.
- Really interesting concept, I'd be very interested to understand the reasoning that the AI tool uses to get to its suggestions, as sometimes they were very different to what I would have done, so it would be useful to know when I was missing something.
- Always difficult as clinical trajectory is associated with uncertainty. very generous use of noradrenaline overall it seemed and this is not without consequence. Potential to help nurse/junior clinician if validation can be confirmed.
- Very interesting to see how Ai is thinking. Made me realise how my human interaction is very much based on a select few parameters, and different layouts are more likely to lead me to potentially miss, or misunderstand some observations. In my own practice, seeing patients and then setting physiological parameters is common. The ability of AI to adjust automatically to meet this would be a huge help in reducing chances of inappropriate drops/ increases of human physiology.
- I found I often disagreed with the AI clinician and agreed with the colleague input
- it showed me my own proclivities to using fluids rather than NA. I'm more comfortable with a 'wet' approach than a dry one. I think this is a reflection of what I have observed in colleagues and seniors over recent years. Similarly, my likelihood to use, or ignore, AI suggestions will - I imagine - be determined more than anything else by senior (sensible in my view) colleagues and their acceptance or derision of the technology. Also, the possible criticism clinicians may feel they open themselves up to if they fail to follow the algorithm.
- Excellent premise. I think this has loads of potential. I guess difficulty is knowing what the 'gold standards' are with regards to fluid/vasopressor therapy. The model has been exposed to more patient 'lab data' than any clinician, but how much does clinical examination and the resulting 'data' play a role in these decisions?
- More explanation/data around the evidence for the categories chosen as most important by the AI facility would give me more faith in following those instructions.
